# *catena*-Poly[[tris­(acetonitrile-κ*N*)praseodymium(III)]tris­(μ-trifluoro­methane­sulfonato-κ^2^ *O*:*O*′)]

**DOI:** 10.1107/S1600536812049525

**Published:** 2012-12-08

**Authors:** Christos Apostolidis, Olaf Walter

**Affiliations:** a European Commission, Joint Research Centre, Institute for Transuranium Elements, Hermann-von-Helmholtz-Platz 1, 76344 Eggenstein-Leopoldshafen, Germany

## Abstract

In the colourless title compound, [Pr(CF_3_O_3_S)_3_(CH_3_CN)_3_]_*n*_, the three trifluoro­methane­sulfonate anions form three bridges *via O*:*O*′-coordination between two Pr^III^ atoms. The structure contains [Pr(NCMe)_3_-μ_2_(OTf)_3_—Pr(NCMe)_3_-μ_2_(OTf)_3_]_*n*_ (NCMe is acetonitrile; OTf is trifluoromethanesulfonate) chains parallel to the *a* axis. The Pr^III^ atom is nine-coordinate in a distorted tricapped trigonal-prismatic environment.

## Related literature

For the isostructural Eu^III^ and U^III^ compounds, see: Tang *et al.* (2011 ▶) and Natrajan *et al.* (2005 ▶), respectively.
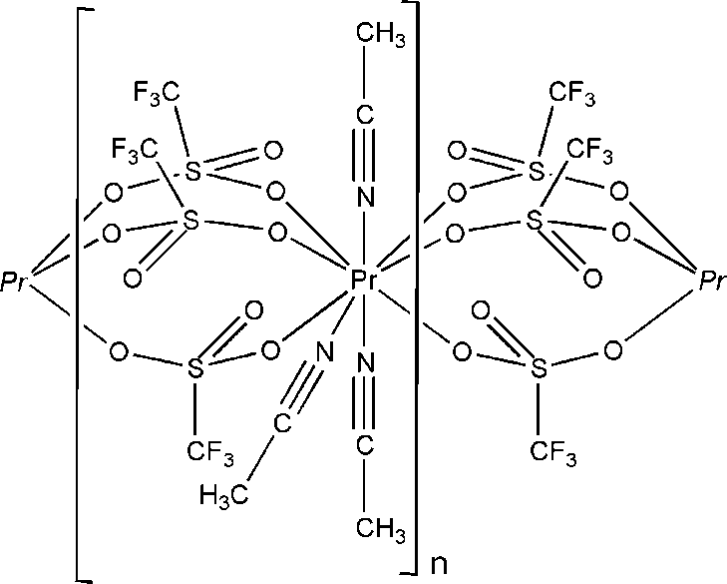


## Experimental

### 

#### Crystal data

[Pr(CF_3_O_3_S)_3_(C_2_H_3_N)_3_]
*M* *_r_* = 711.28
Triclinic, 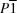

*a* = 5.8044 (6) Å
*b* = 10.5062 (10) Å
*c* = 18.9887 (19) Å
α = 97.307 (1)°
β = 94.163 (1)°
γ = 91.695 (1)°
*V* = 1144.7 (2) Å^3^
*Z* = 2
Mo *K*α radiation
μ = 2.52 mm^−1^
*T* = 103 K
0.24 × 0.02 × 0.01 mm

#### Data collection

Bruker APEXII Quazar diffractometer
Absorption correction: multi-scan (*SADABS*; Bruker, 2001 ▶) *T*_min_ = 0.578, *T*_max_ = 0.978
20544 measured reflections
5251 independent reflections
4674 reflections with *I* > 2σ(*I*)
*R*_int_ = 0.039

#### Refinement

*R*[*F* ^2^ > 2σ(*F* ^2^)] = 0.036
*wR*(*F* ^2^) = 0.076
*S* = 1.16
5251 reflections
311 parameters
H-atom parameters constrained
Δρ_max_ = 1.04 e Å^−3^
Δρ_min_ = −1.15 e Å^−3^

### 

Data collection: *APEX2* (Bruker, 2007 ▶); cell refinement: *SAINT* (Bruker, 2007 ▶); data reduction: *SAINT*; program(s) used to solve structure: *SHELXS97* (Sheldrick, 2008 ▶); program(s) used to refine structure: *SHELXL97* (Sheldrick, 2008 ▶); molecular graphics: *XPMA* (Zsolnai, 1996 ▶) and *ORTEP-3* (Farrugia, 2012 ▶); software used to prepare material for publication: *publCIF* (Westrip, 2010 ▶).

## Supplementary Material

Crystal structure: contains datablock(s) I, global. DOI: 10.1107/S1600536812049525/vn2063sup1.cif

Structure factors: contains datablock(s) I. DOI: 10.1107/S1600536812049525/vn2063Isup2.hkl

Supplementary material file. DOI: 10.1107/S1600536812049525/vn2063Isup3.mol

Additional supplementary materials:  crystallographic information; 3D view; checkCIF report

## Figures and Tables

**Table 1:** Selected bond lengths (Å)

Pr1—O1^i^	2.435 (3)
Pr1—O2	2.464 (3)
Pr1—O4^i^	2.455 (3)
Pr1—O5	2.464 (3)
Pr1—O7	2.459 (3)
Pr1—O8^i^	2.473 (3)
Pr1—N1	2.621 (4)
Pr1—N2	2.648 (4)
Pr1—N3	2.651 (4)

Symmetry code: (i) 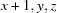.

**Fig. 1.**
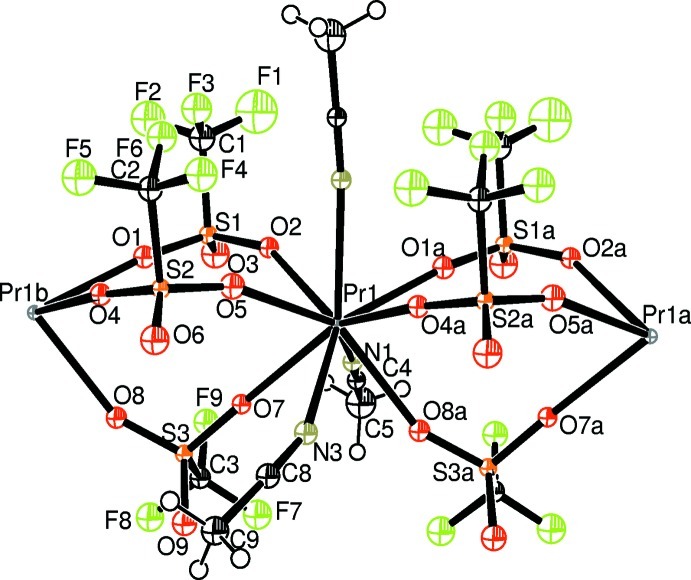
Molecular structure of the title compound; ellipsoids at the 50% probability level (symmetry codes: a = x + 1, y, z; b = x - 1, y, z).

## supplementary crystallographic information

### Comment

The coordination environment of the Pr^III^ atom ion in the title compound can be described as a distorted trigonal tri-capped prism. 6 O atoms are forming the trigonal prismatic environment around the Pr^III^ atom and the three N atoms of the coordinated MeCN solvent molecules are forming the cappings over the rectangular sides of the prism. The metal-O distances in the title compound are with an average of 2.458 (13) Å about 0.05 Å longer than in the corresponding Eu complex (Tang *et al.*, 2011) and 0.04 Å shorter than in its U analogue (Natrajan *et al.*, 2005) reflecting the effects of the lanthanide contraction and also the change from a lanthanide to an actinide ion. The metal-N distances with 2.588 (17) Å for the Eu complex are about 0.05 Å shorter than for the here presented Pr compound with 2.640 (17) Å, whereas the latter are found to be in very good agreement with those in the U complex with 2.651 (14) Å. The ion size increase while changing the metal ion in the complex from Pr to U seems to influence more the shorter metal-O distances than the longer metal-N distances for the solvent molecules forming the cappings of the trigonal prism.

### Experimental

Crystals from the title compound precipitated after a ligand exchange reaction from 103.1 mg (0.13 mmol) [Pr(H_2_O)_9_](OTf)_3_ in 2 ml of MeCN. Crystals in the form of needles suitable for x-ray analysis were obtained by re-crystallization from hot MeCN.

### Refinement

All H atoms were placed on geometrical positions according to the hybridization of the atoms they are bound to. One common isotropic *U* value was used and refined for all hydrogen atoms.

### Crystal data

**Table:**

[Pr(CF_3_O_3_S)_3_(C_2_H_3_N)_3_]	*Z* = 2
*M**_r_* = 711.28	*F*(000) = 688
Triclinic, *P*1	*D*_x_ = 2.064 Mg m^−^^3^
Hall symbol: -P 1	Mo *K*α radiation, λ = 0.71073 Å
*a* = 5.8044 (6) Å	Cell parameters from 9222 reflections
*b* = 10.5062 (10) Å	θ = 2.2–28.1°
*c* = 18.9887 (19) Å	µ = 2.52 mm^−^^1^
α = 97.307 (1)°	*T* = 103 K
β = 94.163 (1)°	Needle, colourless
γ = 91.695 (1)°	0.24 × 0.02 × 0.01 mm
*V* = 1144.7 (2) Å^3^	

### Data collection

**Table:**

Bruker APEXII Quazar diffractometer	5251 independent reflections
Radiation source: fine-focus sealed tube	4674 reflections with *I* > 2σ(*I*)
Graphite monochromator	*R*_int_ = 0.039
Detector resolution: 66 pixels mm^-1^	θ_max_ = 28.2°, θ_min_ = 1.1°
combined ω and φ scans	*h* = −7→7
Absorption correction: multi-scan (*SADABS*; Bruker, 2001)	*k* = −13→13
*T*_min_ = 0.578, *T*_max_ = 0.978	*l* = −25→24
20544 measured reflections	

### Refinement

**Table:**

Refinement on *F*^2^	Primary atom site location: structure-invariant direct methods
Least-squares matrix: full	Secondary atom site location: difference Fourier map
*R*[*F*^2^ > 2σ(*F*^2^)] = 0.036	Hydrogen site location: inferred from neighbouring sites
*wR*(*F*^2^) = 0.076	H-atom parameters constrained
*S* = 1.16	*w* = 1/[σ^2^(*F*_o_^2^) + (0.0169*P*)^2^ + 4.422*P*] where *P* = (*F*_o_^2^ + 2*F*_c_^2^)/3
5251 reflections	(Δ/σ)_max_ = 0.001
311 parameters	Δρ_max_ = 1.04 e Å^−^^3^
0 restraints	Δρ_min_ = −1.15 e Å^−^^3^

### Special details

**Table:**

Geometry. All e.s.d.'s (except the e.s.d. in the dihedral angle between two l.s. planes) are estimated using the full covariance matrix. The cell e.s.d.'s are taken into account individually in the estimation of e.s.d.'s in distances, angles and torsion angles; correlations between e.s.d.'s in cell parameters are only used when they are defined by crystal symmetry. An approximate (isotropic) treatment of cell e.s.d.'s is used for estimating e.s.d.'s involving l.s. planes.
Refinement. Refinement of *F*^2^ against ALL reflections. The weighted *R*-factor *wR* and goodness of fit *S* are based on *F*^2^, conventional *R*-factors *R* are based on *F*, with *F* set to zero for negative *F*^2^. The threshold expression of *F*^2^ > σ(*F*^2^) is used only for calculating *R*-factors(gt) *etc*. and is not relevant to the choice of reflections for refinement. *R*-factors based on *F*^2^ are statistically about twice as large as those based on *F*, and *R*- factors based on ALL data will be even larger.

### Fractional atomic coordinates and isotropic or equivalent isotropic displacement parameters (Å^2^)

**Table:**

	*x*	*y*	*z*	*U*_iso_*/*U*_eq_	
Pr1	1.07535 (4)	0.07978 (2)	0.262548 (13)	0.01276 (7)	
S1	0.56250 (16)	0.18346 (10)	0.15067 (6)	0.0158 (2)	
S2	0.61165 (16)	0.18294 (10)	0.39162 (6)	0.0158 (2)	
S3	0.55261 (16)	−0.16488 (10)	0.24609 (6)	0.0147 (2)	
F1	0.6947 (6)	0.3922 (3)	0.10237 (19)	0.0467 (9)	
F2	0.3309 (6)	0.3778 (3)	0.11712 (19)	0.0450 (9)	
F3	0.5755 (5)	0.4245 (3)	0.20746 (15)	0.0299 (6)	
F4	0.7997 (5)	0.3949 (3)	0.46088 (18)	0.0440 (8)	
F5	0.4258 (5)	0.3828 (3)	0.45649 (18)	0.0422 (8)	
F6	0.5975 (5)	0.4226 (3)	0.36502 (17)	0.0344 (7)	
F7	0.7193 (5)	−0.3223 (3)	0.14700 (16)	0.0320 (7)	
F8	0.3474 (5)	−0.3357 (3)	0.14802 (16)	0.0338 (7)	
F9	0.5120 (4)	−0.1770 (3)	0.10682 (14)	0.0258 (6)	
O1	0.3838 (5)	0.1578 (3)	0.19688 (18)	0.0240 (7)	
O2	0.7937 (5)	0.1773 (3)	0.18415 (17)	0.0216 (7)	
O3	0.5278 (6)	0.1225 (3)	0.07935 (17)	0.0276 (7)	
O4	0.3864 (5)	0.1540 (3)	0.35450 (17)	0.0223 (7)	
O5	0.7984 (5)	0.1725 (3)	0.34527 (17)	0.0230 (7)	
O6	0.6489 (5)	0.1277 (3)	0.45609 (17)	0.0270 (7)	
O7	0.7431 (5)	−0.0746 (3)	0.24309 (17)	0.0208 (7)	
O8	0.3323 (5)	−0.1040 (3)	0.25044 (16)	0.0186 (6)	
O9	0.5924 (5)	−0.2595 (3)	0.29293 (17)	0.0233 (7)	
N1	1.0406 (6)	−0.0433 (4)	0.1330 (2)	0.0225 (8)	
N2	1.1033 (6)	0.3339 (4)	0.2835 (2)	0.0216 (8)	
N3	1.0456 (6)	−0.0546 (4)	0.3708 (2)	0.0214 (8)	
C1	0.5396 (8)	0.3551 (4)	0.1440 (3)	0.0238 (10)	
C2	0.6081 (8)	0.3553 (5)	0.4193 (3)	0.0270 (11)	
C3	0.5309 (7)	−0.2545 (4)	0.1566 (2)	0.0207 (9)	
C4	1.0185 (7)	−0.1019 (5)	0.0786 (3)	0.0219 (10)	
C5	0.9892 (9)	−0.1778 (6)	0.0081 (3)	0.0377 (13)	
H5A	0.9788	−0.2693	0.0134	0.083 (9)*	
H5B	1.1219	−0.1607	−0.0189	0.083 (9)*	
H5C	0.8472	−0.1540	−0.0174	0.083 (9)*	
C6	1.0979 (7)	0.4424 (5)	0.2948 (3)	0.0261 (11)	
C7	1.0879 (10)	0.5816 (5)	0.3096 (5)	0.057 (2)	
H7A	0.9471	0.6098	0.2854	0.083 (9)*	
H7B	1.2236	0.6219	0.2923	0.083 (9)*	
H7C	1.0864	0.6067	0.3611	0.083 (9)*	
C8	0.9551 (7)	−0.1080 (4)	0.4105 (2)	0.0212 (9)	
C9	0.8291 (8)	−0.1738 (5)	0.4598 (3)	0.0283 (11)	
H9A	0.7095	−0.1184	0.4790	0.083 (9)*	
H9B	0.9365	−0.1933	0.4989	0.083 (9)*	
H9C	0.7567	−0.2538	0.4346	0.083 (9)*	

### Atomic displacement parameters (Å^2^)

**Table:**

	*U*^11^	*U*^22^	*U*^33^	*U*^12^	*U*^13^	*U*^23^
Pr1	0.00653 (9)	0.01308 (12)	0.01839 (12)	−0.00110 (7)	0.00174 (7)	0.00089 (8)
S1	0.0118 (4)	0.0162 (5)	0.0198 (5)	−0.0006 (4)	0.0023 (4)	0.0039 (4)
S2	0.0104 (4)	0.0182 (5)	0.0177 (5)	−0.0012 (4)	0.0018 (4)	−0.0015 (4)
S3	0.0101 (4)	0.0124 (5)	0.0215 (5)	−0.0011 (3)	0.0013 (4)	0.0017 (4)
F1	0.069 (2)	0.0256 (17)	0.051 (2)	−0.0047 (15)	0.0294 (18)	0.0160 (16)
F2	0.0451 (19)	0.0280 (17)	0.059 (2)	0.0138 (14)	−0.0205 (16)	0.0072 (16)
F3	0.0326 (15)	0.0204 (15)	0.0351 (17)	0.0005 (11)	0.0012 (12)	−0.0014 (12)
F4	0.0312 (16)	0.0360 (18)	0.055 (2)	−0.0067 (13)	−0.0136 (14)	−0.0178 (16)
F5	0.0357 (16)	0.0319 (18)	0.056 (2)	0.0040 (13)	0.0219 (15)	−0.0165 (15)
F6	0.0276 (15)	0.0199 (15)	0.056 (2)	−0.0004 (11)	0.0017 (13)	0.0051 (14)
F7	0.0307 (15)	0.0266 (16)	0.0381 (17)	0.0115 (12)	0.0087 (12)	−0.0034 (13)
F8	0.0293 (15)	0.0263 (16)	0.0419 (18)	−0.0140 (12)	0.0013 (12)	−0.0068 (13)
F9	0.0236 (13)	0.0315 (16)	0.0222 (14)	0.0012 (11)	0.0006 (10)	0.0031 (12)
O1	0.0164 (14)	0.0217 (17)	0.0349 (19)	−0.0018 (12)	0.0103 (13)	0.0036 (14)
O2	0.0137 (14)	0.0215 (17)	0.0292 (18)	−0.0002 (12)	−0.0021 (12)	0.0045 (14)
O3	0.0307 (17)	0.0279 (19)	0.0228 (18)	−0.0002 (14)	0.0009 (13)	−0.0010 (14)
O4	0.0141 (14)	0.0206 (17)	0.0297 (18)	−0.0033 (12)	−0.0037 (12)	−0.0019 (14)
O5	0.0198 (15)	0.0198 (17)	0.0299 (18)	0.0023 (12)	0.0111 (13)	−0.0002 (14)
O6	0.0243 (16)	0.034 (2)	0.0228 (18)	0.0030 (14)	0.0035 (13)	0.0051 (15)
O7	0.0120 (13)	0.0223 (17)	0.0275 (18)	−0.0090 (11)	0.0001 (12)	0.0034 (14)
O8	0.0146 (14)	0.0171 (16)	0.0242 (17)	0.0023 (11)	0.0031 (11)	0.0010 (13)
O9	0.0208 (15)	0.0204 (17)	0.0304 (19)	−0.0004 (12)	0.0013 (13)	0.0110 (14)
N1	0.0139 (17)	0.029 (2)	0.024 (2)	0.0009 (15)	0.0015 (14)	0.0008 (18)
N2	0.0129 (16)	0.020 (2)	0.032 (2)	−0.0025 (14)	0.0031 (14)	0.0038 (17)
N3	0.0165 (17)	0.022 (2)	0.026 (2)	0.0023 (14)	0.0029 (15)	0.0017 (17)
C1	0.025 (2)	0.018 (2)	0.029 (3)	0.0009 (17)	0.0011 (18)	0.008 (2)
C2	0.018 (2)	0.025 (3)	0.034 (3)	−0.0017 (18)	0.0005 (18)	−0.010 (2)
C3	0.0157 (19)	0.017 (2)	0.027 (3)	−0.0003 (16)	0.0022 (17)	−0.0055 (19)
C4	0.0127 (19)	0.029 (3)	0.024 (3)	0.0004 (17)	0.0024 (16)	0.005 (2)
C5	0.038 (3)	0.051 (4)	0.021 (3)	0.000 (2)	0.002 (2)	−0.006 (2)
C6	0.0120 (19)	0.023 (3)	0.044 (3)	−0.0016 (16)	0.0060 (18)	0.004 (2)
C7	0.032 (3)	0.016 (3)	0.123 (7)	−0.001 (2)	0.022 (3)	0.002 (3)
C8	0.017 (2)	0.022 (2)	0.025 (2)	0.0014 (17)	0.0001 (17)	0.002 (2)
C9	0.022 (2)	0.041 (3)	0.023 (2)	−0.005 (2)	0.0025 (18)	0.010 (2)

### Geometric parameters (Å, º)

**Table:**

Pr1—O1^i^	2.435 (3)	F4—C2	1.340 (5)
Pr1—O2	2.464 (3)	F5—C2	1.333 (5)
Pr1—O4^i^	2.455 (3)	F6—C2	1.320 (6)
Pr1—O5	2.464 (3)	F7—C3	1.333 (5)
Pr1—O7	2.459 (3)	F8—C3	1.332 (5)
Pr1—O8^i^	2.473 (3)	F9—C3	1.324 (5)
Pr1—N1	2.621 (4)	O1—Pr1^ii^	2.435 (3)
Pr1—N2	2.648 (4)	O4—Pr1^ii^	2.455 (3)
Pr1—N3	2.651 (4)	O8—Pr1^ii^	2.473 (3)
S1—O3	1.420 (3)	N1—C4	1.130 (6)
S1—O1	1.447 (3)	N2—C6	1.134 (6)
S1—O2	1.450 (3)	N3—C8	1.142 (6)
S1—C1	1.832 (5)	C4—C5	1.464 (7)
S2—O6	1.426 (3)	C5—H5A	0.9800
S2—O5	1.443 (3)	C5—H5B	0.9800
S2—O4	1.445 (3)	C5—H5C	0.9800
S2—C2	1.822 (5)	C6—C7	1.457 (7)
S3—O9	1.429 (3)	C7—H7A	0.9800
S3—O7	1.444 (3)	C7—H7B	0.9800
S3—O8	1.449 (3)	C7—H7C	0.9800
S3—C3	1.829 (5)	C8—C9	1.457 (6)
F1—C1	1.322 (5)	C9—H9A	0.9800
F2—C1	1.320 (5)	C9—H9B	0.9800
F3—C1	1.326 (5)	C9—H9C	0.9800
			
O1^i^—Pr1—O4^i^	75.59 (11)	O9—S3—C3	105.0 (2)
O1^i^—Pr1—O7	139.23 (11)	O7—S3—C3	102.39 (19)
O4^i^—Pr1—O7	139.22 (11)	O8—S3—C3	103.59 (19)
O1^i^—Pr1—O2	88.88 (10)	S1—O1—Pr1^ii^	170.4 (2)
O4^i^—Pr1—O2	137.29 (10)	S1—O2—Pr1	151.05 (19)
O7—Pr1—O2	75.57 (10)	S2—O4—Pr1^ii^	162.7 (2)
O1^i^—Pr1—O5	137.30 (11)	S2—O5—Pr1	161.1 (2)
O4^i^—Pr1—O5	88.00 (10)	S3—O7—Pr1	169.2 (2)
O7—Pr1—O5	76.01 (10)	S3—O8—Pr1^ii^	155.19 (18)
O2—Pr1—O5	76.90 (11)	C4—N1—Pr1	175.9 (4)
O1^i^—Pr1—O8^i^	77.31 (10)	C6—N2—Pr1	174.3 (3)
O4^i^—Pr1—O8^i^	79.12 (10)	C8—N3—Pr1	156.4 (3)
O7—Pr1—O8^i^	88.37 (10)	F2—C1—F1	109.2 (4)
O2—Pr1—O8^i^	136.43 (10)	F2—C1—F3	108.1 (4)
O5—Pr1—O8^i^	138.43 (10)	F1—C1—F3	108.5 (4)
O1^i^—Pr1—N1	71.04 (11)	F2—C1—S1	110.0 (3)
O4^i^—Pr1—N1	137.12 (11)	F1—C1—S1	109.9 (3)
O7—Pr1—N1	68.20 (11)	F3—C1—S1	111.1 (3)
O2—Pr1—N1	68.43 (11)	F6—C2—F5	107.8 (4)
O5—Pr1—N1	134.88 (11)	F6—C2—F4	107.8 (4)
O8^i^—Pr1—N1	68.00 (11)	F5—C2—F4	108.2 (4)
O1^i^—Pr1—N2	70.21 (11)	F6—C2—S2	112.9 (3)
O4^i^—Pr1—N2	70.03 (11)	F5—C2—S2	110.0 (3)
O7—Pr1—N2	132.10 (10)	F4—C2—S2	110.0 (3)
O2—Pr1—N2	67.28 (11)	F9—C3—F8	108.5 (4)
O5—Pr1—N2	67.15 (11)	F9—C3—F7	108.1 (4)
O8^i^—Pr1—N2	139.51 (10)	F8—C3—F7	108.3 (4)
N1—Pr1—N2	120.35 (12)	F9—C3—S3	111.7 (3)
O1^i^—Pr1—N3	136.38 (11)	F8—C3—S3	110.3 (3)
O4^i^—Pr1—N3	71.01 (11)	F7—C3—S3	109.8 (3)
O7—Pr1—N3	68.24 (11)	N1—C4—C5	179.8 (6)
O2—Pr1—N3	134.74 (10)	C4—C5—H5A	109.5
O5—Pr1—N3	68.85 (11)	C4—C5—H5B	109.5
O8^i^—Pr1—N3	69.59 (11)	H5A—C5—H5B	109.5
N1—Pr1—N3	118.54 (12)	C4—C5—H5C	109.5
N2—Pr1—N3	120.91 (12)	H5A—C5—H5C	109.5
O3—S1—O1	115.7 (2)	H5B—C5—H5C	109.5
O3—S1—O2	115.4 (2)	N2—C6—C7	179.2 (5)
O1—S1—O2	112.91 (19)	C6—C7—H7A	109.5
O3—S1—C1	104.8 (2)	C6—C7—H7B	109.5
O1—S1—C1	103.3 (2)	H7A—C7—H7B	109.5
O2—S1—C1	102.54 (19)	C6—C7—H7C	109.5
O6—S2—O5	115.65 (19)	H7A—C7—H7C	109.5
O6—S2—O4	114.9 (2)	H7B—C7—H7C	109.5
O5—S2—O4	113.45 (19)	N3—C8—C9	177.2 (5)
O6—S2—C2	105.1 (2)	C8—C9—H9A	109.5
O5—S2—C2	102.6 (2)	C8—C9—H9B	109.5
O4—S2—C2	102.99 (19)	H9A—C9—H9B	109.5
O9—S3—O7	115.75 (18)	C8—C9—H9C	109.5
O9—S3—O8	115.15 (19)	H9A—C9—H9C	109.5
O7—S3—O8	112.91 (18)	H9B—C9—H9C	109.5

Symmetry codes: (i) *x*+1, *y*, *z*; (ii) *x*−1, *y*, *z*.
